# Experimental and Meshless Numerical Simulations on the Crack Propagation of Semi-Circular Bending Specimens Containing X-Shaped Fissures Under Three-Point Bending

**DOI:** 10.3390/ma17143547

**Published:** 2024-07-18

**Authors:** Haiying Mao, Cong Hu, Jianfeng Xue, Taicheng Li, Haotian Chang, Zhaoqing Fu, Wenhui Sun, Jieyu Lu, Jing Wang, Shuyang Yu

**Affiliations:** 1 School of Civil and Architectural Engineering, Guangxi University of Science and Technology, Liuzhou 545006, China; maohaiying16@163.com; 2 China Renewable Energy Engineering Institute, Beijing 100120, China; xuejf@creei.cn (J.X.); changht@creei.cn (H.C.); fuzq@creei.cn (Z.F.); 3 School of Transportation and Civil Engineering, Nantong University, Nantong 226019, China; 2133110304@stmail.ntu.edu.cn (W.S.); 19906298072@163.com (J.L.); 2133110334@stmail.ntu.edu.cn (J.W.)

**Keywords:** X-shaped fissures, SCB specimens, numerical simulations, crack propagation

## Abstract

Cracks in rock and concrete have a great adverse effect on the stability of engineering structures; however, there are few studies on X-shaped fissures which widely exist in rock and concrete structures. Based on this background, three-point bending fracture tests of SCB specimens containing X-shaped fissures are carried out. The momentum equations in the SPH method are improved, and the crack propagations of SCB specimens under three-point bending are simulated. The results show that cracks grow simply along the vertical direction in the sample with no X-shaped fissures, and the existence of an X-shaped fissure changes the crack growth path and final failure modes of the SCB samples. The crack propagation simulation results are consistent with the experimental results, which verifies the rationality of the improved SPH method. The load–displacement curves mainly present three typical stages: the initial compaction stage, linear elastic deformation stage, and failure stage. The peak load decreases first then increases with an increase in eccentricity. With an increase in X-shaped fissure length and decrease in X-shaped fissure angle, the peak load decreases. The damage counts remain at 0 at the initial loading stage, corresponding to the initial compaction stage and the linear elastic deformation stage, and increase sharply at the later loading stage, corresponding to the failure stage, which is consistent with the experimental results. The influence mechanisms of X-shaped fissures on the crack propagation paths are discussed; the existence of different X-shaped fissure morphologies aggravate the tensile stress concentration at specific positions, leading to different crack propagation modes in the experiments. The research results can provide a certain reference for understanding the failure mechanisms of engineering structures containing X-shaped fissures and promote the applications of the SPH method into the simulations of cross-fissure crack propagations.

## 1. Introduction

**Figure 1:**
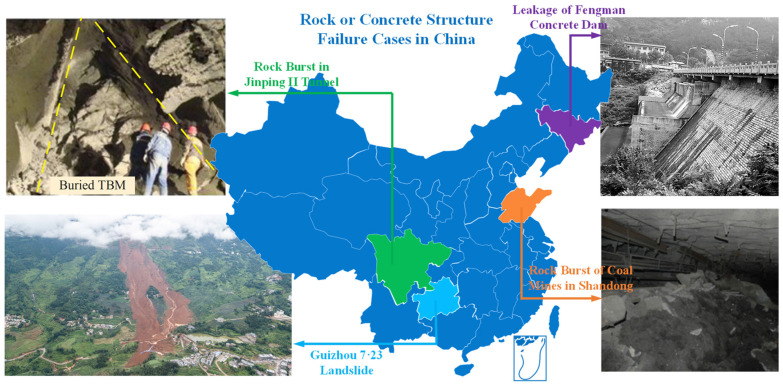
Typical failure cases of rock or concrete structures in China [2,3,4,5].

As an inherent property of materials, fissures widely exist in rock and concrete structures. For rock engineering, crack propagation and penetration are very easy to occur under human activities; for example, cracks will propagate when excavations are carried out in underground caverns, mines, slopes, and related projects, thus reducing the structure stability [1]. In fact, the deformation and failure processes of rock masses are essentially the processes of crack initiation, expansion, interaction and penetration under engineering disturbances. Therefore, complex fissure morphologies change the stress states in rock masses and further affect the failure modes of engineering rock structures. For concrete structures, the influences of internal fissures mainly manifest as (1) reducing the concrete strength; (2) causing the leakage or spalling of the concrete cover; (3) leading to steel corrosion; (4) accelerating concrete carbonization, etc. Finally, they will lead to the deteriorations of concrete quality, shortening the service life, and even contributing to structural instabilities. Crack propagation in engineering materials will lead to the failure of engineering structures. For example: a high-level landslide occurred in Chagou Formation, Pingdi Village, Jichang Town, Shuicheng County, Guizhou Province on 23 July 2019, killing 43 people and leaving 9 missing [2]; the diversion tunnel of the Jinping II hydropower station experienced a strong rock burst during the excavation processes [3]. The concrete gravity dam of Fengman Hydropower Station suffered freeze–thaw damage during operations, resulting in seepage in the reservoir [4]. Serious rock burst accidents occurred in the underground deep wells of Suncun Coal Mine and Liangbaosi Coal Mine [5], as shown in Figure 1. It can be inferred that internal fissures in rock or concrete engineering structures can lead to the occurrence of engineering disasters, seriously threatening the life, safety, and properties of people in disaster areas. Therefore, investigating the crack propagation and evolution laws of brittle materials such as rock and concrete will provide certain references for preventing engineering disasters and understanding the disaster mechanisms. 

Scholars have conducted extensive experimental, theoretical, and numerical simulations on the crack propagation laws of solid materials and their internal mechanisms. Experimental research can provide the most intuitive and realistic representations of material crack distribution morphologies; for example, Brace et al. [6] conducted uniaxial experimental research on brittle rock specimens containing one single-inclined fissure and found that cracks propagated along the direction of approximately 70° of the original crack surface. Bobet et al. [7] analyzed the crack initiation, propagation, and penetration mechanisms of pre-cracked rock specimens with different geometric distributions, and found that the crack penetration modes were mainly influenced by loading conditions and pre-cracked geometric distributions. Zhang et al. [8] conducted a series of uniaxial compression tests on specimens containing double fissures with different inclination angles based on acoustic emission and digital image correlation technology. Zhou et al. [9] studied the strength, failure, and crack evolutions of samples containing cross fissures under shear loading conditions. Semi-circular bending (SCB) specimens are widely used in the research of fracture behaviors of brittle materials due to their simple geometry, manufacturing processes, and experimental operations. For example: Wu et al. [10] used SCB specimens to study the cracking behavior of reflection cracks, and obtained the variation rules of fracture energy; Xu et al. [11] studied the effects of notch width and notch tip angle on the fracture toughness of SCB specimens; and Zhou et al. [12] investigated the variation rules of mode I/III mixed-fracture toughness of SCB specimens with different prefabricated fissure angles under different loading speeds. However, the existing experimental research mainly focuses on the non-cross fissures, and the fracture mechanical behaviors of X-shaped fissures under semi-circular bending conditions are rarely involved. Theoretical research is based on the physical phenomenon of rock fracture, summarizing and extracting the quantitative mathematical expressions of rock properties, which can reflect the general laws of rock fracture. For example: Wang et al. [13] proposed the maximum tensile stress criterion based on *J*-integral theory and applied it to the traditional discontinuous deformation analysis; and Chen et al. [14] established a method to quantitatively describe the relationships between stress and cracks in concrete. However, the limitation of theoretical research is that it can only deal with mathematical problems under simple loads and boundary conditions. For problems with multiple fissures, cross fissures, complex loads, or geometric shapes, theoretical research can not give quantitative mathematical expressions.

As the “third method” of scientific research, numerical simulation not only gets rid of the “circle” of mathematical equations in theoretical research, but also can reflect the internal mechanisms of material deformation and failure that can not be exhibited in traditional experimental research. Through numerical calculation and image display, the purpose of studying engineering problems can then be achieved. Therefore, numerical simulation is widely used in the simulations of crack propagations. The finite element method (FEM) is one of the earliest methods used to study fracture mechanics; for example, Han et al. [15] simulated the shear behaviors of rock-like materials containing fissures and holes and divided the shear processes into four typical stages based on the FEM-CZM method; Yang et al. [16] used the extended finite element method (XFEM) to study the effects of fracture orientation, matrix stiffness, and confining stress on the equivalent permeability of fractured porous media; and Jia et al. [17] proposed a method using the FE-FEM to simulate the dynamic fracture of static cracks in linear elastic solids. However, the FEM needs mesh re-generation and stress mapping when dealing with discontinuous features during crack propagation simulations, which can easily cause calculation failures and reduce the calculation accuracy. The discrete element method (DEM) is a meshless method which discretizes the computational domain into a series of particles. The DEM can reflect the complex interactions by using various contact models, so it can easily simulate the crack propagation processes without being restricted by mesh re-divisions existing in the traditional FEM. For example: Yang et al. [18] used a two-dimensional particle flow program (PFC2D) to simulate the strength, deformation, and crack evolutions of sandstone containing a single elliptical cavity under uniaxial compression; Li et al. [19] studied the fracture processes of granite rock samples and the possible factors affecting crack initiation, propagation, and consolidation by using the particle-based finite–discrete element method (GB-FDEM); and Deng et al. [20] used the finite–discrete element method (FDEM) to study the failure mechanisms of layered rock masses. However, the DEM has many meso-parameters which have no practical physical meanings, so complex parameter calibrations are needed before calculation. The mesh-free methods also include the lattice element method [21] and Smoothed Particle Hydrodynamics. The Smoothed Particle Hydrodynamics (SPH) method is a pure Lagrange meshless method that combines the advantages of the FEM and DEM. This feature can conveniently solve the discontinuous problems of crack propagation simulations. Nevertheless, the applications of SPH into rock fracture mechanics are relatively rare at present; only Zhou’s groups have proposed the GPD method based on SPH [22,23,24,25,26,27,28,29,30], which has great advantages in simulating rock crack propagation. However, the SPH method is seldom involved in the simulations of rock cross-fissure interactions.

In view of the previous shortcomings, SCB samples containing X-shaped fissures were prepared using brittle transparent materials (PMMA). Three-point bending tests were conducted on SCB samples with different X-shaped fissure properties, and the crack propagation morphology as well as the load–displacement laws were obtained. The experimental results obtained from homogeneous PMMA (polymethyl methacrylate) have lower dispersion, which exclude the influences of the heterogeneity existing in rock and concrete materials, and can better explore the influences of different forms of X-shaped fissures on rock crack propagation paths. In addition, the improved SPH method is applied in simulating the crack propagation processes of SCB samples, and the simulation results are compared with previous experimental results to verify the rationality of the method. Finally, the fracture mechanisms of X-shaped fissures are discussed. The research results can provide some references for the understanding of the fracture laws of rock masses containing X-shaped fissures, and promote the applications of the SPH method into rock fracture mechanics.

## 2. Experiment Preparation

**Figure 2:**
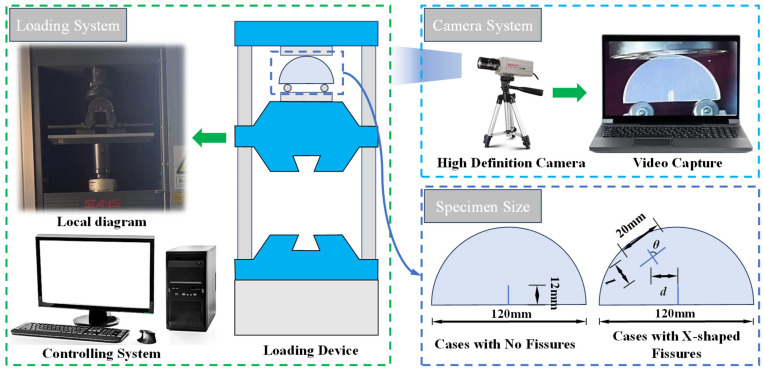
Experimental devices and test schemes.

The experimental system is mainly divided into two parts: a loading system and camera system, as shown in Figure 2. The loading system is mainly composed of a loading device and controlling system. The loading device applies CMT5205, whose loading force value ranges from 0 to 200 kN. The controlling system uses a desktop computer, and the loading rate is controlled by servo software. The loading rate in our experiment was set to be 0.5 mm/min with uniaxial compression. The camera system captures the real-time crack propagation of the specimen during the loading processes through the HD camera.

As pre-existing fissures have great impacts on rock strength [31,32], SCB specimens were utilized in our experiment with a semi-circular shape and a diameter of 120 mm. One guide fissure was prefabricated in the sample, with a length of 12 mm. The X-shaped fissure was also prefabricated in the specimen; the length of one X-shaped fissure was 20 mm, and the other was defined as *l*. The angle between two X-shaped fissures is defined as *θ*. In order to explore the effects of different X-shaped fissure angles *θ*, lengths *l*, and eccentricities *d* on the failure modes of SCB samples, different experimental schemes with various X-shaped fissure properties were designed and the details are listed in Table 1.

## 3. Numerical Treatments of Material Failure in SPH

### 3.1. SPH Theories

**Figure 3:**
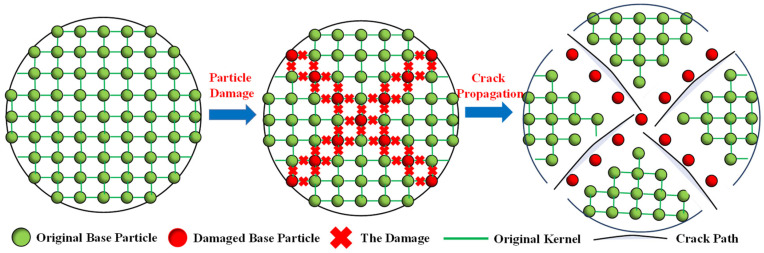
Numerical treatments of particle failure in SPH.

As the basic theories of SPH have been illustrated in many of the previous studies [30], we have only listed the core points of SPH failure equations. SPH has been widely applied in solid mechanics [33,34]. In order to characterize the failure processes of particles in SPH, the “activation state” coefficient *η* is introduced, and the improved smoothing kernel function A considering particle “activation” and “failure” is defined, which can consider each SPH particle to be “live” or “dead”. The numerical treatments are shown in Figure 3, and the smoothing kernel function A can be expressed as the product of the “activation state” coefficient *η* and the traditional smoothing kernel function *W* [25]:

(1) $$A(x-x^{\prime },\;h)\;=\eta \cdot W(x-x^{\prime },\;h)$$

By substituting Equation (5) into SPH’s momentum Equation (4), the SPH momentum equation considering particle “activation” and “failure” can be obtained [25]:

(2) $$\frac{dv_{i}^{\alpha }}{dt}=\sum_{j\in S}m_{j}(\frac{\sigma_{ij}^{\alpha \beta }}{\rho_{i}^{2}}+\frac{\sigma_{ij}^{\alpha \beta }}{\rho_{j}^{2}}+T_{ij})\partial A_{ij,\beta }+\sum_{j\in D}m_{j}(\frac{\sigma_{ij}^{\alpha \beta }}{\rho_{i}^{2}}+\frac{\sigma_{d}^{\alpha \beta }}{\rho_{j}^{2}}+T_{ij})\partial A_{ij,\beta }$$

The determination of the fracture criterion is the prerequisite for accurately simulating material failure. In this section, the widely used maximum tensile stress criterion is utilized. When the tensile stress of the particle reaches this tensile strength, then the particle is damaged. The expression of this criterion can be expressed as:

(3) $$\sigma_{1}=\sigma_{t}$$

where *σ_t_* is the strength parameter of the particle and represents the tensile strength; *σ*_1_ is the maximum principal stress of the particle.

### 3.2. Establishment of Numerical Models

**Figure 4:**
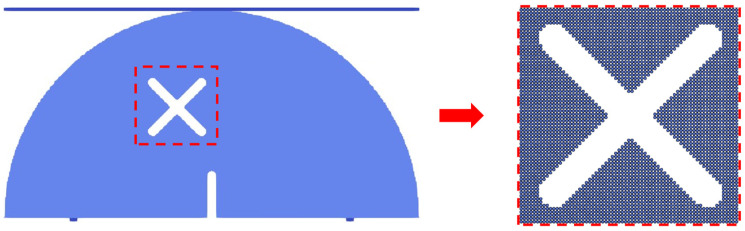
SPH numerical model and particle divisions.

The numerical model is shown in Figure 4 (taking test scheme B3 as an example). The model sizes were consistent with those used in the experiment, and the whole model was divided into 61,342 particles. The numerical model applied the displacement loading mode. In order to accelerate the calculation processes, the loading rate was set to be 0.005 m/s, and the calculation time step Δ*t* was set to be 5 × 10^−9^ s. The numerical parameters were as follows: elastic modulus *E* = 17 GPa, Poisson’s ratio *μ* = 0.2, and tensile strength *σ_t_* = 1 MPa. The mass of the sample was excluded and the inertial effects were ignored in the simulations.

## 4. Experimental and Numerical Results

### 4.1. Fracture Morphology of SCB Samples Containing X-Shaped Fissures

**Figure 5:**
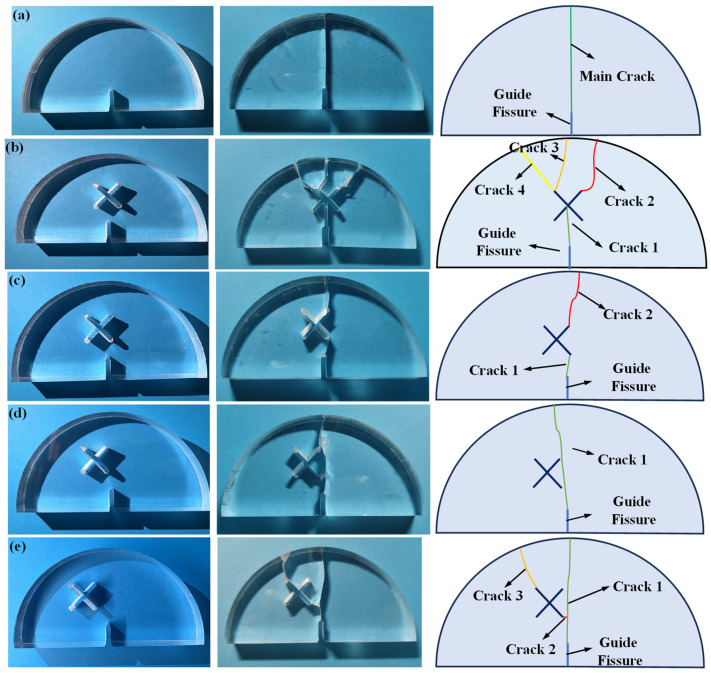
SCB failure morphology in scheme A and scheme B. (**a**) Scheme A: no X-shaped fissures; (**b**) Scheme B1: *d* = 0 mm; (**c**) Scheme B2: *d* = 5 mm; (**d**) Scheme B3: *d* = 10 mm; (**e**) Scheme B4: *d* = 15 mm.

Figure 5 shows the fracture morphology of the SCB samples in scheme A and scheme B1 ~ B4. As can be seen: for the circumstance of scheme A, the main crack initiates from the guide fissure tip and propagates along the vertical direction to the top of the specimen, causing the specimen A to break into two symmetrical halves. The existence of X-shaped fissures greatly influences the crack growth paths and the fracture morphology of SCB samples. For the circumstance of *d* = 0 mm, crack 1 initiates from the guide fissure tip. When crack 1 overlaps with the center of the X-shaped fissure, three secondary cracks are generated from the upper end of the X-shaped fissure, which are denoted as crack 2, crack 3 and crack 4, respectively. Crack 3 and crack 4 are generated from the left end of the X-shaped fissure, and crack 2 is generated from the right end of the X-shaped fissure. Finally, crack 2, crack 3, and crack 4 expand to the top of the SCB sample and lead to failure. For the circumstance of *d* = 5 mm, crack 1 firstly initiates from the guide fissure tip, and then overlaps with the lower right tip of the X-shaped fissure, then crack 2 is generated from the upper right tip of the X-shaped fissure. Finally, crack 2 propagates through the top of the SCB specimen and leads to failure. For the circumstance of *d* = 10 mm, crack 1 initiates from the guide fissure tip, and propagates towards the X-shaped fissure due to the “attraction” effect. Different from other conditions, the X-shaped fissure does not produce extra secondary cracks, and crack 1 finally propagates through the SCB sample. For the circumstance of *d* = 15 mm, crack 1 initiates from the guide fissure tip, and the crack growth path is less affected by the X-shaped fissure, extending almost vertically. During the propagation processes of crack 1, crack 2 is formed by bonding with the lower left tip of the X-shaped fissure, and crack 3 is also generated from the upper left end of the X-shaped fissure. Finally, crack 1 and crack 3 expand to the top of the SCB sample at the same time, causing the sample failure.

**Figure 6:**
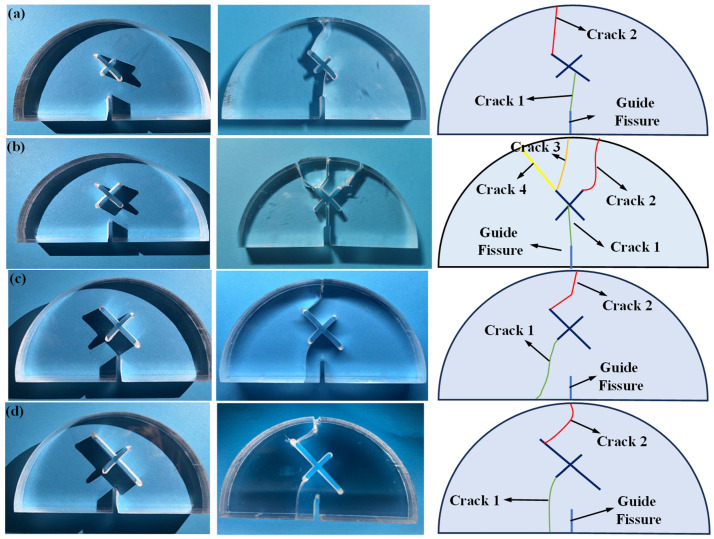
SCB failure morphology in scheme C. (**a**) Scheme C1: *l* = 10 mm; (**b**) Scheme C2: *l* = 20 mm; (**c**) Scheme C3: *l* = 30 mm; (**d**) Scheme C4: *l* = 40 mm.

Figure 6 shows the fracture morphologies of SCB samples with different X-shaped fissure lengths *l*. As can be seen, for the circumstance of *l* = 10 mm, crack 1 firstly initiates from the guide fissure tip, and overlaps with the lower end of the longer X-shaped fissure. Then, crack 2 is generated at the upper tip of the longer X-shaped fissure, which propagates to the top of the specimen and causes the failure. For the circumstance of *l* = 20 mm, due to its symmetry, crack 1 initiates from the guide fissure tip and propagates to the center of the X-shaped fissure. Then, crack 2, crack 3, and crack 4 are generated from the upper end of the X-shaped fissure and expand to the top of the SCB specimen. For the circumstance of *l* = 30 mm and *l* = 40 mm, crack 1 initiates from the lower part of the specimen and overlaps with the shorter X-shaped fissure, then crack 2 propagates from the upper tip of the longer X-shaped fissure to the specimen top, leading to the failure.

**Figure 7:**
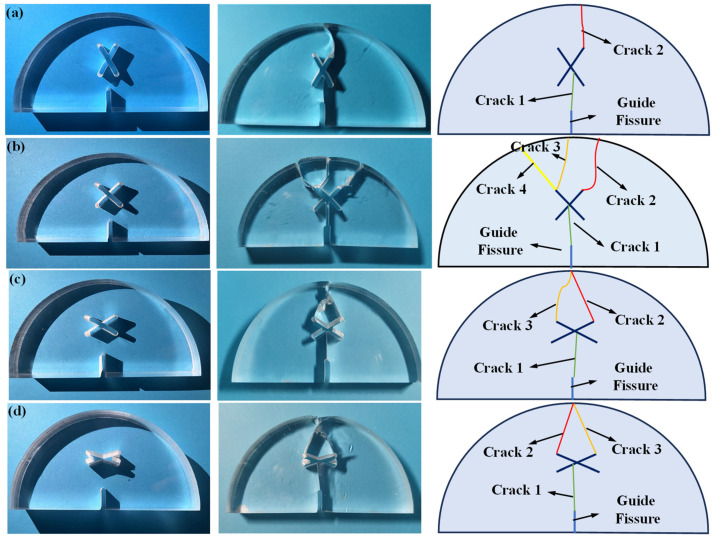
SCB failure morphology in scheme D. (**a**) Scheme D1: *θ* = 60°; (**b**) Scheme D2: *θ* = 90°; (**c**) Scheme D3: *θ* = 120°; (**d**) Scheme D4: *θ* = 150°.

Figure 7 shows the fracture morphologies of SCB samples with different X-shaped fissure angles. As can be seen, for the circumstance of *θ* = 60°, crack 1 firstly initiates from the guide fissure tip, and propagates approximately to the center of the X-shaped fissure. Then, crack 2 is generated from the upper right of the X-shaped fissure and extends to the top of the specimen, resulting in the specimen failure. For the circumstance of *θ* = 90°, crack 1 initiates from the guide fissure tip, and overlaps with the center of the X-shaped fissure. Subsequently, three secondary cracks (crack 1, crack 2, and crack 3) are generated from the upper end of the X-shaped fissure, which expand to the specimen top and cause the failure. For the circumstance of *θ* = 120° and *θ* = 150°, crack 1 initiates from the guide fissure tip and overlaps with the center of the X-shaped fissure. Then, crack 2 and crack 3 are generated at the at the upper end of the X-shaped fissure. Finally, crack 2 and crack 3 expand to the top of the sample, leading to the specimen failure. It is worth noting that crack 2 and crack 3 converge at the upper loading point, making the fracture morphology of the entire sample symmetrical.

### 4.2. Simulation Results of SCB Specimens Containing X-Shaped Fissures

**Figure 8:**
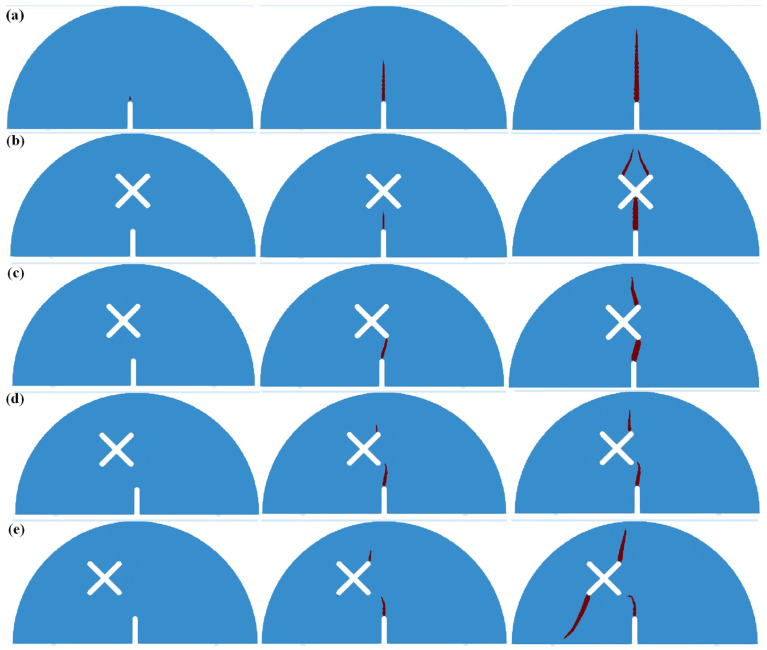
Numerical results of SCB specimen crack propagation under scheme A and scheme B. (**a**) Scheme A: no X-shaped fissures; (**b**) Scheme B1: *d* = 0 mm; (**c**) Scheme B2: *d* = 5 mm; (**d**) Scheme B3: *d* = 10 mm; (**e**) Scheme B4: *d* = 15 mm.

Figure 8 shows the simulation results of SCB specimens under scheme A and scheme B. As can be seen from the figure, for the circumstance of scheme A, the crack firstly initiates from the guide fissure tip and propagates vertically, eventually penetrating the model and causing the model failure. For the circumstance of *d* = 0 mm, a crack initiating from the guide fissure propagates to the center of the X-shaped fissure, then the upper two ends of the X-shaped fissure produce two cracks, which expand to the model top and lead to the failure. For the circumstance of *d* = 5 mm and *d* = 10 mm, a crack generated by the guide fissure tip propagates to the lower right end of the X-shaped fissure, then the crack initiates from the upper right end of the X-shaped fissure and propagates to the loading point, resulting in the model failure; For the circumstance of *d* = 15 mm, the crack generated from the guide fissure tip gradually propagates to the lower right end of the X-shaped fissure, and then the crack initiating from the upper right end of the X-shaped fissure propagates to the direction of the loading point. Finally, the crack initiating from the lower left end of the X-shaped fissure extends to the lower part of the model, and the crack at the right upper side propagates to the loading point, thus leading to the model failure. The numerical results are highly consistent with the experimental results.

**Figure 9:**
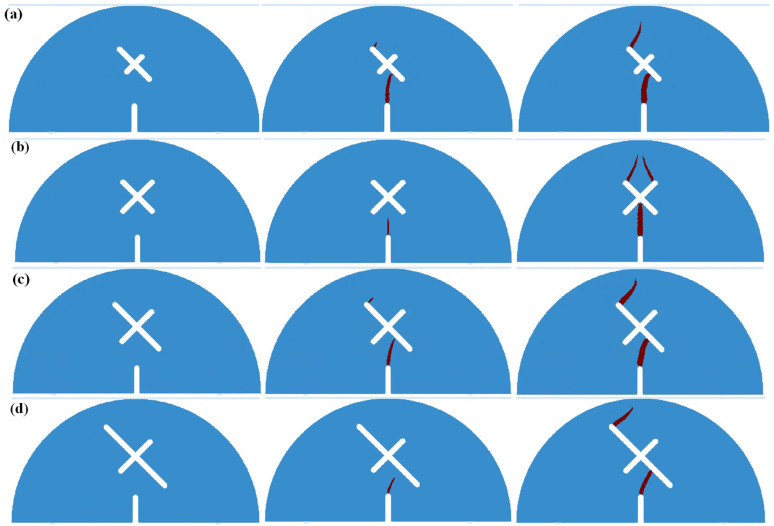
Numerical results of SCB specimen crack propagation under scheme C. (**a**) Scheme C1: *l* = 10 mm; (**b**) Scheme C2: *l* = 20 mm; (**c**) Scheme C3: *l* = 30 mm; (**d**) Scheme C4: *l* = 40 mm.

Figure 9 shows the simulation results of SCB specimens under scheme C. As can be seen: when the length of the two X-shaped fissures is not equal (*l* = 10 mm, *l* = 30 mm, *l* = 40 mm), the crack initiating from the guide fissure firstly connects with the lower end of the long X-shaped fissure, then the crack propagation occurs at the upper end of the long X-shaped fissure, and its direction is towards the direction of the upper loading point. When the length of the two X-shaped fissures is equal, the crack generated from the guide fissure propagates to the center of the X-shaped fissure, and then the cracks at the upper side of the X-shaped fissures symmetrically propagate to the loading point of the model. The numerical results are consistent with the experimental results.

**Figure 10:**
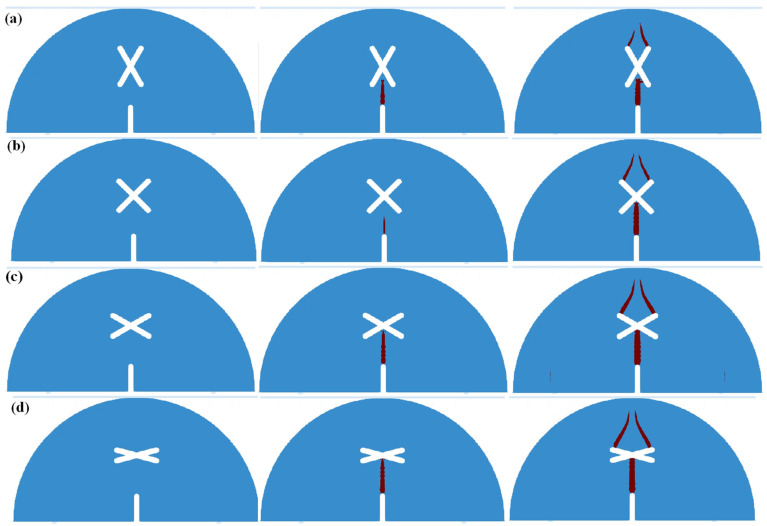
Numerical results of SCB specimen crack propagation under scheme D. (**a**) Scheme D1: *θ* = 60°; (**b**) Scheme D2: *θ* = 90°; (**c**) Scheme D3: *θ* = 120°; (**d**) Scheme D4: *θ* = 150°.

Figure 10 shows the simulation results of SCB specimens under scheme D. As can be seen, due to the symmetry of the model, crack propagation firstly occurs at the guide fissure tip, and then extends to the center of the X-shaped fissure. After that, cracks initiate from the two upper ends of the X-shaped fissure and extend symmetrically to the loading point of the model. The numerical results are highly consistent with the experimental results.

### 4.3. Effects of X-Shaped Fissure Properties on SCB Specimen Strength and Damage Counts

**Figure 11:**
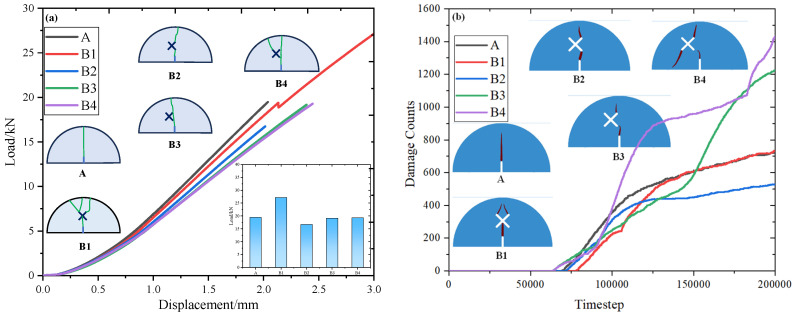

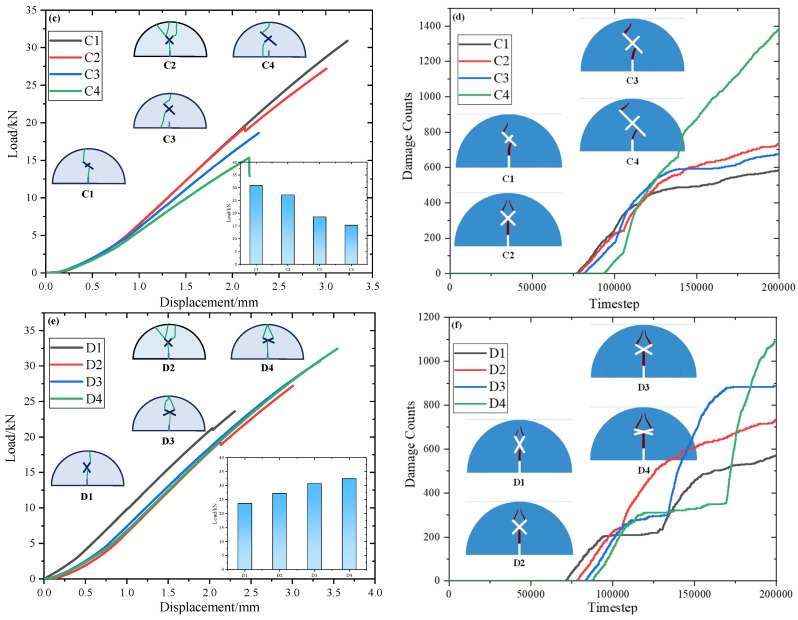
Displacement–load curves and damage count curves of SCB specimens. (**a**) Displacement–load curves of scheme A and scheme B; (**b**) damage count curves of scheme A and scheme B; (**c**) displacement–load curves of scheme C; (**d**) damage count curves of scheme C; (**e**) displacement–load curves of scheme D; (**f**) damage count curves of scheme D.

Figure 11 shows the effects of different X-shaped fissure angles *θ*, lengths *l*, and eccentricities *d* on the strength and damage counts of SCB specimens. As can be seen, the load–displacement curves of the SCB specimens can be divided into three stages: the first stage is the initial compaction stage, in which the load–displacement curve is concave, indicating that the cracks inside the samples are gradually compacted; the second stage is the linear elastic deformation stage, in which the load and displacement increase linearly and no damage occurs inside the SCB specimens; and the final stage is the failure stage, and the load drops sharply at this stage.

For scheme A and scheme B1, the existence of X-shaped fissures increases the peak strength of the specimen, which may be due to the fact that the X-shaped fissure increases the flexibility of the SCB sample, leading to an increase in the deformation of the sample, and thus increasing the peak strength. Meanwhile, with an increase in eccentricity *d*, the peak load of SCB specimens decreases first and then increases. When eccentricity *d* = 5 mm, the peak load reaches the minimum. This is because the lower right X-fissure tip is closest to the guide fissure tip in this scheme, which will make the stress field around the guide fissure easier to be influenced by the X-shaped fissure. Thus, the specimen is easier to be damaged and the peak load is the smallest. The damage counts remain 0 at the beginning of the loading, indicating that there is no crack growth inside the model at this time, corresponding to the initial compaction and linear elastic deformation stage in the experiment. However, after reaching the crack initiation load, the damage counts increase sharply, and the variation rules of damage counts are consistent with those of the experiments. For the condition with different X-shaped fissure lengths *l*: With an increase in X-shaped fissure length *l*, the peak strength of SCB specimens decreases, indicating that the mechanical properties of the specimen are greatly affected by the lower end of the long X-shaped fissure. The final numerical damage counts increase with an increase in the fissure length *l*, indicating that the damage degree of the model is greater, which is consistent with the experimental results. For the condition with different X-shaped fissure angles *θ*, the peak strength increases with an increase in θ; this is because an increase in *θ* leads to an increase in the distance between the lower end of the X-shaped fissure and the guide fissure, thus reducing the interactions between the two parts and increasing the peak strength of the sample. The final numerical damage counts also increase with an increase in the X-shaped fissure angle *θ*, which is consistent with the variations of the specimen peak strength.

## 5. Discussions

### 5.1. Effects of X-Shaped Fissures on Crack Propagation Paths

**Figure 12:**
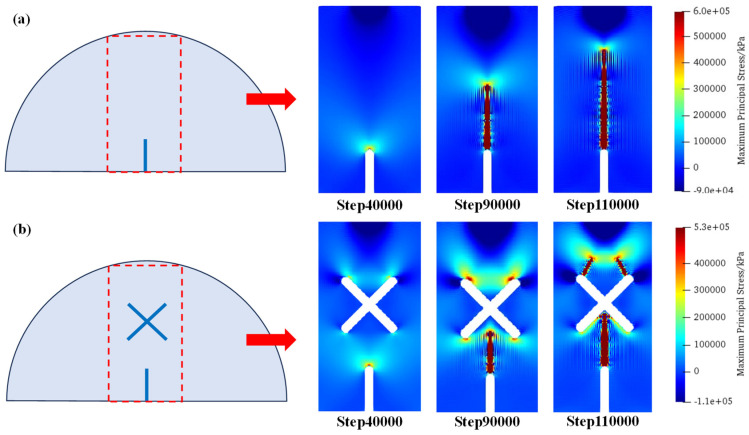
Maximum principal stress distributions of scheme A and scheme B1. (**a**) Scheme A; (**b**) Scheme B1.

Figure 12 shows the distributions of maximum principal stress under scheme A and scheme B1 at step 40,000, step 90,000, and step 110,000. As can be seen, for the circumstance of scheme A (no X-shaped fissures), the tensile stress concentrates at the guide fissure tips; therefore, a crack initiates from the guide fissure, and propagates towards the vertical direction, which has a relatively simple propagation path. For the circumstance of scheme B1 (an X-shaped fissure exists): During the initial loading stage, tensile stress concentrates at the guide fissure tip. However, after the crack initiates and propagates, tensile stress concentrates on the upper sides of the X-shaped fissures, where the cracks propagate symmetrically to the loading point, thus forming a different crack propagation mode compared with scheme A.

### 5.2. Asymmetric Crack Propagation Mechanisms under the Case of Eccentric X-Shaped Fissures

**Figure 13:**
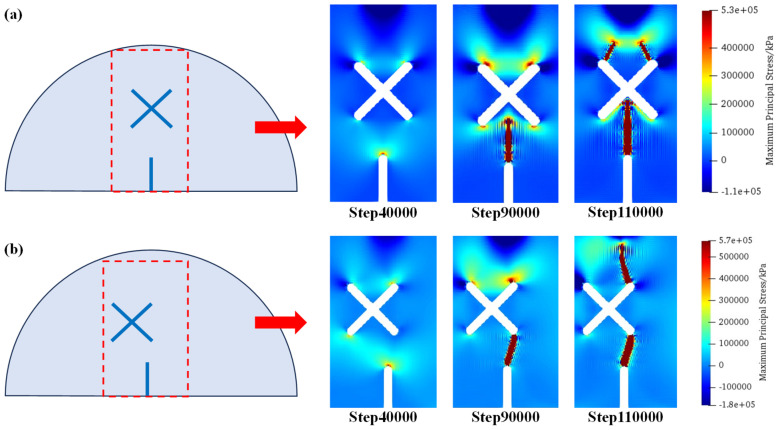
Maximum principal stress distributions of scheme B1 and scheme B2. (**a**) Scheme B1; (**b**) Scheme B2.

Figure 13 shows the distributions of maximum principal stress under scheme B1 and scheme B2 at step 40,000, step 90,000, and step 110,000. As can be seen, different from the symmetric fracture morphology in scheme B1, due to the existence of eccentricity, cracks initiating from the guide fissure firstly connect with the X-shaped fissure. Then, the tensile stress concentration of the upper right tip of the X-shaped fissure becomes greater; therefore, cracks are generated from the upper right tips of the X-shaped fissures and propagate to the loading point of the model.

### 5.3. Crack Propagation Mechanisms of X-Shaped Fissure with Unequal Length

**Figure 14:**
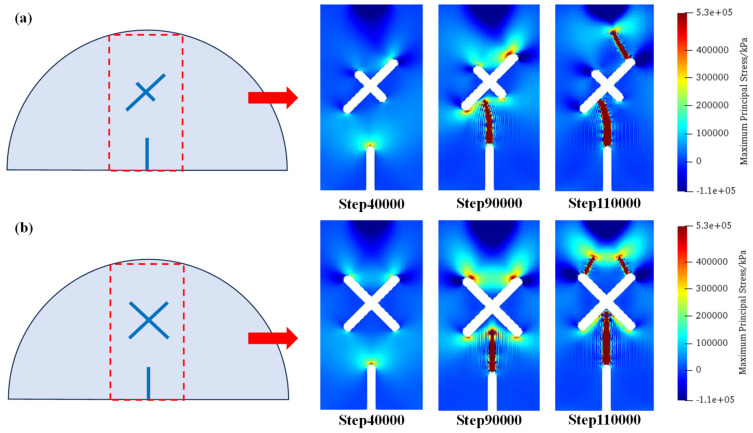
Maximum principal stress distributions of scheme C1 and scheme C2. (**a**) Scheme C1; (**b**) Scheme C2.

Figure 14 shows the distributions of maximum principal stress under scheme C1 and C2 at step 40,000, step 90,000, and step 110,000. As can be seen, compared with the conditions where the X-shaped fissure lengths are equal, the unequal fissure length intensifies the concentration of tensile stress at the upper end of the long fissure. Therefore, after the cracks initiate from the guide fissure and connect with the long X-shaped fissure, the upper tip of the X-shaped fissure produces crack propagation, which exhibits an asymmetric fracture morphology in scheme C1. However, in the circumstance of equal-length X-shaped fissures (scheme C2), the cracks are symmetrically generated from the two upper tips of the X-shaped fissures.

### 5.4. Research Prospects in Experiments and Simulations

In our work, the improved SPH method is utilized to simulate the progressive failure processes of X-shaped fissures. Compared with the FEM, SPH does not need to re-divide the mesh grids, which can simulate the complex rock failure processes; compared with the DEM, SPH does not have many microscopic parameters. We used PMMA material to prepare the rock-like samples. The PMMA specimens were free of cracks before the tests began except for the X-shaped fissure. The selection of PMMA was due to its transparent nature, meaning that we could directly observe the fracture morphologies. In addition, the experimental results obtained from the homogeneous PMMA have lower dispersion, which exclude the influences of the heterogeneity existing in rock and concrete materials, and can better explore the influences of different forms of X-shaped fissures on rock crack propagation paths. However, in actual engineering, 3D cracks widely exist, so future works should focus on 3D conditions both in experiments and numerical simulations.

## 6. Conclusions

(1) SCB (semi-circular bending) specimens containing X-shaped fissures were prepared, and three-point bending tests were carried out. The crack growth paths of specimens with no X-shaped fissures are relatively simple; the cracks extend vertically from the guide fissure. The existence of X-shaped fissures greatly alters the crack growth path and the final fracture morphology.
(2) The momentum equation in SPH (Smoothed Particle Hydrodynamics) was improved and the “activation state” coefficient *η* was defined to simulate the brittle fracture characteristics of solids. The crack propagation processes of SCB samples containing X-shaped fissures were simulated. The simulation results were consistent with the experimental results, which verify the rationality of the improved method; the improved SPH method can be well applied to simulations of rock fractures.
(3) The load–displacement curves of the SCB (semi-circular bending) specimens present three stages: an initial compaction stage, linear elastic deformation stage, and failure stage. The peak strength of SCB (semi-circular bending) specimens decrease first then increase with an increase in eccentricity *d*, and decrease with an increase in X-shaped fissure length as well as a decrease in X-shaped fissure angle. The damage counts remain 0 at the initial loading stage, corresponding to the initial compaction stage and the linear elastic deformation stage, but increase sharply at the later loading stage, corresponding to the failure stage, which is consistent with the experimental results.
(4) The influence mechanisms of X-shaped fissures on the fracture modes of the SCB (semi-circular bending) specimens are discussed. The existence of an X-shaped fissure intensifies the concentrations of tensile stress on its upper sides, and thus alters the vertical propagation modes existing in the circumstance with no X-shaped fissures. The eccentricity and different length of X-shaped fissures aggravate the tensile stress concentration at the unilateral tip, thus forming an asymmetric crack propagation pattern.

## Figures and Tables

**Table 1:** Experimental schemes.

Number	Test Scheme	Number	Test Scheme
A	No X-shaped fissures	C3	*l* = 30 mm
B1	*d* = 0 mm	C4	*l* = 40 mm
B2	*d* = 5 mm	D1	*θ* = 60°
B3	*d* = 10 mm	D2	*θ* = 90°
B4	*d* = 15 mm	D3	*θ* = 120°
C1	*l* = 10 mm	D4	*θ* = 150°
C2	*l* = 20 mm		

## Data Availability

The original contributions presented in the study are included in the article, further inquiries can be directed to the corresponding author.
